# Reduced *Dicer *expression in the cord blood of infants admitted with severe respiratory syncytial virus disease

**DOI:** 10.1186/1471-2334-11-59

**Published:** 2011-03-08

**Authors:** Christopher S Inchley, Tonje Sonerud, Hans O Fjærli, Britt Nakstad

**Affiliations:** 1 Department of Pediatrics, Akershus University Hospital, Lørenskog, Norway; 2 Institute of Clinical Medicine, Akershus University Hospital, University of Oslo, Lørenskog, Norway; 3 EpiGen Institute, Akershus University hospital, Lørenskog, Norway

## Abstract

**Background:**

Respiratory syncytial virus (RSV) is one of the most important causes of pediatric hospital admissions in the developed world. The ribonuclease Dicer is an important regulator of gene expression and cellular function via RNA interference, and may also have anti-viral functions. A previous microarray analysis of the cord blood of 5 patients with RSV disease suggested downregulation of *Dicer*. In order to further investigate whether reduced *Dicer *expression can predispose newborns to RSV disease, we have analyzed the gene expression of *Dicer *in the cord blood of 37 infants with confirmed RSV disease.

**Methods:**

The cord blood of 2108 newborns was collected. 51 had a positive nasopharyngeal aspirate for RSV <1 year, and were grouped according to disease severity. 37 had sufficient cord blood RNA of good quality. *Dicer *gene expression was assessed by qPCR analysis of cord blood using a TaqMan low-density array and compared to control infants who did not present with RSV disease using the Mann-Whitney test.

**Results:**

There was significant downregulation of *Dicer *in the severe disease group: relative quantity 0.69 (95% CI: 0.56 - 0.87), p = 0.002. There was no significant downregulation in the mild disease group.

**Conclusions:**

We demonstrate reduced *Dicer *expression in the cord blood of infants with severe RSV disease, prior to RSV exposure. We theorize that this may predispose to RSV disease by disruption of leukocyte gene regulation or direct anti-viral RNA interference mechanisms.

## Background

Bronchiolitis and other lower respiratory tract diseases are amongst the most common causes of pediatric admissions [1,2]. In epidemiological studies the most important pathogen causing bronchiolitis has consistently been respiratory syncytial virus (RSV) [1,3-7]. The yearly epidemics of RSV lead to a significant increase in admissions to pediatric wards across the globe during the winter and spring months. Infantile RSV bronchiolitis is associated with later development of asthma in childhood [8], and is therefore a major cause of ongoing disease burden to patients and significant health costs to society [5].

69% of US children are infected with RSV in the first year of life, and almost all by the age of two years [6]. The majority are asymptomatic or have only mild symptoms. International studies estimate the annual incidence of RSV bronchiolitis requiring hospital admission to 22 - 31/1000 amongst infants < 1 year [2,6,9,10].

Why so few children exposed to RSV should develop symptoms requiring hospital admission is yet to be adequately explained, although research has provided important clues in the last 10 years. Some genetic associations with RSV infection have already been described [11-15] and predisposition to RSV bronchiolitis is likely to be multifactorial. Increased knowledge about the pathophysiology of bronchiolitis and predisposing factors will aid researchers in the development of preventative measures and therapies for bronchiolitis [16,17].

Dicer is an RNase III enzyme that produces micro RNA (miRNA) sequences by cleaving nuclear derived pre-miRNA. miRNA interferes with gene expression by binding to complementary mRNA, facilitating mRNA degradation and preventing mRNA translation into protein. This mechanism is called RNA interference (RNAi), and is an important post-transcriptional regulator of gene expression [18,19]. There is good evidence to suggest that RNAi also has a direct anti-viral function. Cellular derived miRNA has specific antiviral effects, interfering with viral gene expression [20-22]. Dicer can also cleave long genomic viral dsRNA sequences into short interfering RNA (siRNA). siRNA can thus be virally derived and have perfect sequence specificity for viral mRNA. While this is a specific anti-viral mechanism in plants and invertebrates, there is currently no evidence to suggest that this is true for mammals, in which the interferon system is more important in viral defense [20,22]. However, synthesized siRNA tailored to specific viruses has been shown to have significant anti-viral effects in humans, in an interferon-independent manner [23,24].

We have previously investigated gene expression by microarray analysis of the cord blood of 5 infants who later developed RSV bronchiolitis [25]. Unpublished results of this study include a tendency to downregulation of *Dicer *in these infants. Our hypothesis is that reduced *Dicer *expression at birth predisposes newborns to RSV disease, and to investigate this we have analyzed *Dicer *expression in the cord blood of 37 infants with confirmed RSV infection.

## Methods

### Collection of cord samples

The Akershus Birth Cohort Biobank was established between January 2003 and February 2004 [25]. From a total of 3500 births at our hospital, the cord blood of 2108 infants was collected. Samples were collected into PaxGene RNA collection tubes (PreAnalytiX), and EDTA tubes. EDTA tubes were centrifuged and a cellular layer with the intention of later DNA analysis was removed. All samples were stored at -80°C. The study was approved by the Regional Norwegian Ethics Committee and we have informed, written maternal consent.

### Identification of RSV infection

On clinical examination in our pediatric emergency unit, nasopharyngeal aspirates (NPAs) were taken routinely in all patients with suspected viral respiratory disease. NPAs were taken by deep nasal suctioning followed by the aspiration of 2 ml viral transport medium and analysis with an RSV rapid antigen test (Abbott TestPack RSV, Abbott laboratories) [26]. If negative, the NPA was analyzed for RSV by multiplex RT-PCR (Prodesse, Inc) [27]. All virological tests were conducted at the Department of Microbiology, Akershus University Hospital.

### Patient identification and clinical information

Patients were defined as those with a positive rapid antigen test or PCR of their NPA for RSV before 1 year of age. To identify these infants, we cross-referenced our cohort with the hospital's microbiological database. Patient medical records were reviewed and clinical data pertaining to the episode of RSV disease retrieved. 17 healthy controls were randomly selected from the cohort of 2108 infants, and their medical records appraised for respiratory or other disease, or testing for RSV in the first 3 years of life. Patients and controls were excluded if they had conditions predisposing to RSV bronchiolitis (anatomical anomalies including congenital heart disease and cleft palate; prematurity before week 34; chronic lung disease; known or suspected genetic or neurological deficits, including Down's syndrome and hypotonia) [9,28,29] or conditions which could significantly alter gene expression at birth (perinatal viral or bacterial infection; small for gestational age, defined as < 10^th ^percentile for weight). Controls were also excluded if they moved from our hospital's population area, were admitted to hospital with respiratory diseases (including bacterial or viral infections, asthma, or other chronic lung conditions), or were positive for RSV before 3 years of age. In order to investigate differences in gestational or delivery factors that might alter gene expression, we reviewed maternal medical records for delivery method, birth weight, Apgar score and placental weight.

6 patients were treated for RSV by their general practitioner (GP), and were not admitted to hospital. Similarly, most of the controls had not been admitted to hospital other than for orthopedic conditions. We do not have clinical data for these patients and controls other than age at which they were tested, the fact that they were not admitted to our hospital, and birth data. We have confirmed that they were not admitted for treatment at other pediatric units in our region by contacting the archives unit at each hospital.

### Disease definition and classification

We defined RSV disease as a positive NPA for RSV irrespective of symptoms. The patients were categorized into mild or severe RSV disease according to a pre-determined algorithm (table 1). The data most consistently recorded in our patients' medical records were use of respiratory support, supplemental oxygen, feeding tubes, intravenous fluids, and the pediatrician's assessment of respiratory effort, including severity of retractions. The disease categorization algorithm was constructed using these data. Importantly, liberal admission practice meant that a number of neonates with mild disease were admitted for observation, and we wanted to ensure that these infants were not classified with severe disease simply on the basis of their admission to hospital. Investigators were blinded to patient Dicer status when determining disease classification. Patients tested for RSV by their GPs, but not referred for admission at this or other nearby hospitals, were considered to have mild disease since referral was presumably not necessary.

**Table 1:** Patient classification algorithm

SEVERE DISEASE	MILD DISEASE
Respiratory Support (CPAP or Mechanical Ventilation)	Not admitted
Apnea related to respiratory exhaustion	Upper respiratory disease only
Oxygen requirement	Mild or no Dyspnea^1^
Cardiovascular compromise^2^	
Feeding tube or intravenous fluids	
Significant Dyspnea^1^	

Note: Patients are classified as having severe or mild RSV disease if they display one of the characteristics in the respective columns. In the event a patient had characteristics from both columns, the patient was classified with severe disease. CPAP: Continuous positive airways pressure.

^1 ^Pediatrician's assessment of respiratory effort.

^2 ^Defined as mottled, grey appearance with pulse > 170, reduced consciousness and clinical response to intravenous bolus of 0.9% saline.

### Preparation of samples for analysis and RNA quality

Total RNA was extracted using PAXgene blood RNA isolation kit (PreAnalytiX) with DNase treatment according to the manufacturer's instructions. The quantity of total RNA was assessed using the NanoDrop ND-1000 Spectrophotometer (NanoDrop Technologies). RNA quality was assessed by the RNA integrity number (RIN), determined using the Agilent 2100 Bioanalyzer with RNA 6000 nanochips (Agilent Technologies). RNA purity was good, with a mean 260/280 ratio 2.05 (SD 0.04; range1.9-2.1); mean 260/230 ratio 1.99 (SD 0.2; range1.4-2.3); and mean RIN 7.8 (SD 0.7; range 4.7 - 9.2). A No-RT control experiment showed high amounts of genomic DNA in our RNA samples prior to conversion to cDNA. To avoid detection of genomic DNA, we selected a probe that spans an exon junction (DICER1-Hs00229023_m1, Applied Biosystems).

### Selection of reference gene

A defective TLDA card sealer resulted in a number of wells not sealing correctly during the qPCR experiment, and there was inadequate amplification in all samples. This affected the 3 selected reference genes, but not *Dicer*. The qPCR experiment was run simultaneously with another gene expression investigation including *ICAM2 *(probe: ICAM2-Hs00168384_m1, Applied Biosystems). Mean *ICAM2 *non-normalized cycle threshold (Ct) was 25.1 (SD 0.43) for the control group, 25.2 (SD 0.5) for the mild RSV group and 25.1 (SD 0.45) for the severe RSV group. Importantly, the Mann-Whitney test revealed no significant differences in *ICAM2 *Ct values between the patient and control groups (p = 0.987). We were thus able to identify *ICAM2 *as a suitable reference gene, and all samples were normalized against *ICAM2*.

### qPCR analysis

The patient and control samples were prepared according to standard guidelines for qPCR experiments (Applied Biosystems): 1 μg total RNA was reverse transcribed using the High Capacity RNA to cDNA Master Mix (Part Number 4390777, Applied Biosystems) and cDNA was added to high molecular grade water to a total volume of 52.5 μl and stored at -20°C. 52.5 μl TaqMan Gene Expression Master Mix was added to the cDNA sample before loading 100 μl to each LDA fill port. Loading, centrifuging and sealing of the TaqMan array was done according to the manufacturer's instructions. The plate was run on the ABI PRISM 7900 HT with TaqMan Array thermal cycling block and the thermal profile: 50°C for 2 min, 94.5°C for 10 min and 45 cycles at 97°C for 30 seconds and 59.7°C for 1 min. ABI Prism SDS2.3 software and RQ Manager 1.2 (Applied Biosystems) was used to process qPCR results.

There was amplification of *Dicer *mRNA in 16 of 17 control samples and in all patient samples. Mean non-normalized *Dicer *Ct = 27.1 (SD 0.71; range 3.3). *Dicer *Ct values were normalized against the reference gene, and then calibrated against the median of the control group, according to the ΔΔCt method [30,31]. The ΔΔCt scale is a 1/log2 scale: doubling the quantity of mRNA in a sample will result in a reduction of the ΔΔCt by 1. Relative quantities of mRNA were therefore calculated using a (2^-ΔΔCt^) transformation.

### Protein analysis

The cellular samples from our cohort were tested for Dicer by Western blot analysis in a selection of samples from our cohort (method described below). We were unable to demonstrate Dicer in our samples. To investigate whether our samples were corrupted, we repeated the procedure in new cord blood samples from infants born June 2010, in adult blood samples and in protein isolated from a colorectal adenocarcinoma cell line, WiDr (ATCC Number CCL-218, LGC Standards).

New cord and adult blood samples were collected in EDTA tubes, centrifuged and the WBC layer sampled. Contaminating red blood cells were treated with FACS lysing solution before centrifugation, wash and a new centrifugation to pellet the WBCs. WiDr cell monolayers were harvested by scraping into PBS stored at 4°C and centrifuged at 3,000 g at 4°C for 5 min. All samples were stored at -80°C.

Proteins were isolated using Mammalian Protein Extraction Reagent (M-PER, Thermo Fisher Scientific). Halt Protease Inhibitor Cocktail, Halt Phosphatase Inhibitor Cocktail (WiDr cells) and EDTA Solution (Thermo Fisher Scientific), were added to the M-PER just before use.

100 μl lysis buffer per well (9,6 cm^2^) was used for the WiDr cell line and homogenates were rotated for 60 min at 4°C and centrifuged (15,000 g for 30 min at 4°C). We used 500 μl lysis buffer per 100 μl Cohort sample and 100 μl lysis buffer on the WBC pellet from adult and new cord samples. Samples were shaken at 750 rpm on an Eppendorf thermomixer for 60 min at 4°C and centrifuged at 14,000 g for 30 min at 4°C. The supernatant was transferred to a new tube for analysis. The extracted protein from new cord blood samples was vacuum centrifuged to achieve sufficient total protein concentrations. 200 μl of some of the cohort samples were sonicated at 10% amplitude on the Branson digital sonifier for 10 sec. Total protein was determined by proteinA280 on the NanoDrop ND-1000 Spectrophotometer. Samples were stored at -80°C.

Western blot analysis for Dicer was performed using 2 Dicer antibodies (Cell Signaling Technologies, product number 3363; and Abcam, product number ab14601) in separate experiments. In the qPCR analysis, our mRNA probe has affinity for the 2 major Dicer transcript variants [GenBank: NM_030621 and NM_177438], and 4 alternative splice variants [GenBank: AB_028449, AB_023145, AJ_132261, and BC_150287] [32]. Our antibodies have specificity for the 2 major variants and all alternative variants except for BC_150287.

Samples were denatured at 95°C for 5 min. Gel loading quantities were: 50 μg WiDr protein; 50 μg adult WBC protein; 50 μg and 150 μg new cord WBC protein and cohort protein. The samples were resolved in 4-20% Linear gradient SDS-PAGE, Criterion Tris-HCl gels (BioRad) and immunoblotted onto PVDF membranes (GE Healthcare). Membranes were blocked in 5% non-fat dried milk in 1x Tris Buffered Saline containing 0.1% Tween20 (1x TBS-T) (BioRad) at room temperature for 1 h and incubated overnight at 4°C with Dicer primary antibody in 1x TBS-T. After washing, the membranes were incubated with secondary antibody in 5% non-fat dried milk in 1x TBS-T for 1 h at room temperature. The blots were visualised by ECL Plus Western blotting detection system (GE Healthcare) according to the supplier's instructions. Membranes were visualised on the LAS-3000 mini (Fujifilm Corporation).

We were unable to demonstrate Dicer in our cohort samples, the new cord blood samples or adult blood samples. Dicer was clearly demonstrated in the WiDr cells.

### Statistical analysis

A power analysis of our previous microarray results [25] indicated a need for 11 individuals in each group in order to achieve a power of 80%. qPCR results were analyzed using the Mann-Whitney test. The median difference between groups was calculated using the Hodges-Lehmann estimator, first calculated for the normally distributed ΔΔCt values, and then converted to relative quantities. For graphical presentation, ΔΔCt values were assigned a negative value so that a lower value represents less *Dicer *mRNA. Patient clinical characteristics were analyzed using Student's t-test and ANOVA for normally distributed parameters, the Mann-Whitney test for non-normally distributed parameters, and the chi-square and Fisher's exact test for categorical parameters. Statistical analyses were carried out using PASWstatistics 17.0 and Minitab 15.0 statistical software. Using a Bonferroni correction to allow for other qPCR experiments carried out concurrently with this investigation, we considered p < 0.005 to be statistically significant for differences in *Dicer *expression, p < 0.025 to be significant for epidemiological characteristics, and p < 0.05 to be significant for clinical characteristics.

## Results

We identified 52 infants from our cohort who tested positive for RSV before 1 year of age. One had cleft palate and was excluded; otherwise there were no patients who met exclusion criteria. Due to use of RNA in previous experiments, 37 had sufficient RNA for analysis. 20 were classified with severe RSV disease and 17 with mild RSV disease. 4 infants with mild disease included in the final analysis were diagnosed by their GP and not sent to hospital. The clinical findings for all identified patients are summarized in tables 2 - 3. There were no significant differences in clinical characteristics between the 14 patients not included, and the 37 included in the qPCR experiment (data not shown).

**Table 2:** Epidemiological and birth data for control, mild RSV and severe RSV groups

	CONTROL		MILD DISEASE		SEVERE DISEASE		**Sig**.
Number	16		17		20		-
Median age on admission (IQR), months	-		3	(2 - 4)	2.5	(1.00 - 5.75)	p = 0.66^1^
Male: Female ratio	8 : 8		9 : 8		10 : 10		p = 0.98^2^
Vaginal Delivery:							
Non-Instrumental	14	82%	12	71%	17	85%	
Instrumental	2	12%	2	12%	1	5%	
Caesarian Section:							p = 0.34^3^
Elective	0	0%	1	6%	2	10%	
Emergency	0	0%	2	12%	0	0%	
Mean Gestational Age (S.D.), weeks	39.6	(1.5)	39.1	(2.1)	39.3	(1.5)	p = 0.54^4^
Mean Birth Weight (S.D.), grams	3628	(590)	3910	(815)	3617	(517)	p = 0.88^4^
Mean placental weight (S.D.), grams	629	(140)	832	(228)	721	(199)	p = 0.008^5^
1 min Apgar < 8	0	(0%)	3	(18%)	1	(5%)	p = 0.14^3^
5 min Apgar < 8	0	(0%)	1	(6%)	0	(0%)	p = 0.34^3^

Note: There was a higher placental weight in the mild disease group compared to controls, but not in the severe disease group. Otherwise, there were no significant differences in gestational or delivery factors that might affect gene expression. Patients in mild and severe groups were ill at similar ages. ^1^Mann-Whitney test; ^2^Fischer's exact test; ^3^chi-square test; ^4^ANOVA.

^5 ^For mild disease vs. control p = 0.008; for severe disease vs. control, p = 0.205 (Student's t-test)

**Table 3:** Clinical features and diagnoses of 37 infants with confirmed RSV disease.

	MILD DISEASE		SEVERE DISEASE		**Sig**.
Number	17		20		-

CLINICAL FEATURES:					
Significant Dyspnea^1^	0	(0%)	17	(85%)	-
Apnea	0	(0%)	2	(10%)	-
Cardiovascular Compromise	0	(0%)	2	(10%)	-
Highest pCO2, mean (S.D.), kPa	5.83	(0.93)	6.70	(1.88)	p = 0.088^2^
Lowest O_2 _saturation; mean (S.D.), %	98	(2.4)	93	(5.9)	p = 0.005^2^
Highest Respiratory Rate; Mean (S.D.),/min	40	(9.7)	50	(8.3)	p = 0.003^2^
Pulse on admission; mean (S.D.),/min	153	(18)	155	(18)	p = 0.806^2^
Weight on admission; mean (S.D.), grams	6470	(1613)	6640	(1910)	p = 0.82^2^
Duration of symptoms on admission; mean (S.D.), days	4.5	(3.4)	4.1	(1.3)	p = 0.62^2^
Length of stay; median (IQR), days	2	(2 - 4)	5	(3 - 5)	p = 0.001^3^

INTERVENTIONS					

CPAP/Ventilator	0	(0%)	0	(0%)	-
Supplemental Oxygen	0	(0%)	8	(40%)	-
Supplemental Fluids					
Intravenous	0	(0%)	2	(10%)	-
Feeding Tube	0	(0%)	3	(15%)	-

CLINICAL DIAGNOSIS:^5^					

Bronchiolitis	12	(71%)	20	(100%)	
Pneumonia	1	(6%)	2	(10%)	
Atelectasis	0	(0%)	2	(10%)	p = 0.407^4^
URTI	1	(6%)	0	(0%)	
Uncertain Diagnosis^6^	4	(24%)	0	(0%)	-

Note: There was a statistically significant reduction in oxygen saturation, increase in respiratory rate and longer length of stay in the severe disease group, and a tendency to higher pCO2. Clinical features, interventions and diagnoses incorporated in the algorithm for disease severity were not analyzed statistically. pCO2: capillary partial pressure of carbon dioxide; URTI = Upper respiratory tract infection.

^1 ^Pediatrician's assessment of respiratory effort.

^2 ^Student's t-test; ^3^Mann-Whitney test; ^4^chi-square test.

^5 ^Some patients received more than one diagnosis, e.g.: bronchiolitis and atelectasis.

^6 ^4 patients tested for RSV by GPs, but not referred to hospital, received an uncertain diagnosis. An inquiry of hospital archives reveals that these patients have not been admitted at other pediatric clinics in our region.

### Dicer Gene Expression

**Figure 1:**
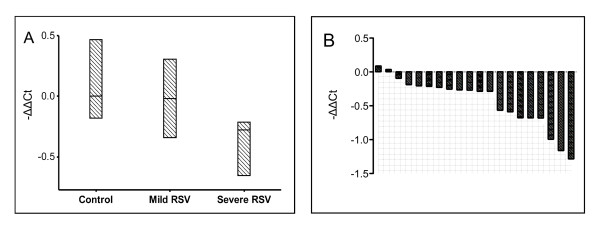
***Dicer *gene expression in cord blood**. A: *Dicer *cord blood gene expression in control and patient groups. Median values with 95% CI. p = 0.002 for severe RSV disease vs. control. B: *Dicer *expression in the cord blood of 20 patients with severe RSV disease, compared to the median of the control group. A negative value represents reduced expression.

There was significant downregulation of *Dicer *in the severe disease group (p = 0.002). There was no significant downregulation in the mild disease group (p = 0.48). Results are demonstrated in table 4 and figure 1. When comparing the severe disease group to the mild disease group, there was a tendency to significance (p = 0.034), when corrected by Bonferroni's method.

**Table 4:** Relative quantities of *Dicer *mRNA in 17 infants with mild and 20 infants with severe RSV disease, compared to 16 controls

	RELATIVE QUANTITY (95% CI)		Sig.
Mild RSV disease	0.89	(0.67 - 1.23)	p = 0.48^1^
Severe RSV disease	0.69	(0.56 - 0.87)	p = 0.002^1^

Note: The median difference and confidence intervals between patient and control groups was calculated using the Hodges-Lehmann estimator for ΔΔCt values, and a 2^-ΔΔCt ^transformation to generate relative quantities. Statistical analysis was done using the Mann-Whitney test. There was significant downregulation of *Dicer *in the severe RSV group but not in the mild RSV group.

^1^Mann-Whitney test

## Discussion

In this experiment we find significantly reduced levels of *Dicer *mRNA in the cord blood of infants who later develop severe RSV disease when compared to controls. This result was not apparent in infants who developed mild RSV disease. The severe disease group tended towards reduced *Dicer *mRNA when compared to the mild disease group. In our cohort, none of the patients with RSV disease required mechanical ventilation or CPAP, the most severe form of the disease. Thus, our findings reflect on the more usual group of patients with significant dyspnea requiring hospital admission but not mechanical respiratory support. There was a larger placental weight in the mild disease group compared to control. There is no evidence in the literature to suggest that this difference would affect our results significantly, and by experience a larger placental weight would be of benefit in otherwise healthy children. Otherwise, we did not identify any differences between the groups at birth that might affect gene expression. There were significant variations in oxygen saturation, respiratory rate and length of stay between the mild and severe RSV groups, and a tendency to increased pCO2 in the severe group, supporting the legitimacy of our disease classification algorithm. To our knowledge this is the first experiment specifically investigating the role of Dicer in RSV disease.

Our selection of controls and classification into mild or severe disease was retrospective and required some assumptions about patients who had not been examined at our hospital for respiratory disease. This includes all the controls and 4 of those with mild disease tested for RSV by their GP, but not referred to hospital. This introduces the possibility of misclassification by several mechanisms: controls may have had RSV disease but not been tested, or may not have visited their GP at all; 4 infants not sent to hospital were assumed to have mild disease, but may have had severe disease; we rely on accurate completion of medical records; there may have been inter-observer variation in the pediatrician's assessment of the severity of dyspnea. Given that 69% of children are infected with RSV in the first year of life [6], it is in fact likely that a number of the controls had RSV disease, but only had mild symptoms (e.g.: rhinitis). Therefore, it is not surprising that we do not show a difference between mild and control groups. Increasing the number of control infants included in the analysis would have increased the power of the study and therefore the chance of discovering a difference between control and mild groups. However, we feel that the groups were so similar that any difference is not likely to be clinically relevant. Patients with unrecognized severe disease included in the control or mild disease group may increase the risk of type 1 or type 2 errors when considering the severe disease group. However, given our experience with the patient population and GPs in our area, we consider that the chance that infants with severe disease would not visit their GP or be referred for admission is small. Altogether, we had a low number of patients in our analysis. Increasing the size of the birth cohort may have increased the total number of patients, the power of the study and the chance of discovering a significant difference between mild and severe disease groups.

Dicer protein was below detection level by Western blot analysis of cord samples from our cohort. This seems remarkable given that the qPCR experiment showed high amounts of *Dicer *mRNA. Our antibodies are also specific for proteins translated from the commonly occurring splice variants identifiable by our mRNA. We corroborated our primary antibodies and Western blot technique by demonstration of Dicer in colorectal cancer cells. We encountered the same phenomenon in new cord and adult peripheral blood leukocyte samples, so degradation of Dicer protein in our cohort samples seems an unlikely explanation. Dicer is essential for, and miRNA profiles are well described in, normal leukocyte function, development and proliferation [33]. We can therefore not explain why Dicer protein was not detectable in our samples. To our knowledge, Dicer has not previously been demonstrated by Western blot analysis in human leukocytes. One could speculate that peripheral blood leukocytes contain other splice variants of Dicer detected by our mRNA probe but not by our antibodies; that leukocyte Dicer is bound to other proteins and therefore not detected in our blots; or that a different Dicer protein is active in leukocytes, but we find no literary reports to confirm this. It may be that *Dicer *mRNA is not transcribed to Dicer protein or the protein is rapidly degraded once produced, but given the necessity for Dicer in leukocytes, this seems unlikely. We can, however, not exclude variations in post-transcriptional modification, and we are unable to measure whether there are differences in Dicer protein level between patients and controls. Thus we cannot verify that the difference we see in *Dicer *mRNA level reflects a real difference in Dicer protein level in our cohort. However, several articles investigating *Dicer *gene expression in cancer have found a good correlation between *Dicer *mRNA level and Dicer protein level [34-36], and *Dicer *mRNA level as a proxy for Dicer protein level therefore seems reasonable.

In cord blood, the main source of mRNA should be nucleated cells, primarily myeloid and lymphoid leukocytes. The functional effect of *Dicer *downregulation in these cells is likely to be two-fold: disrupted cellular function, and reduced direct anti-viral activity. In murine models, absence of Dicer leads to considerable disruption of cell function in T-lymphocytes, natural killer cells and Langerhans cells [37-40]. In alveolar cell cultures, influenza virus mediated *Dicer *downregulation coincides with significantly accelerated cell death [21]. In humans, reduced *Dicer *mRNA levels have been associated with hepatocellular carcinoma, invasive epithelial ovarian cancer and metastatic breast cancer [34-36], and it is likely that disruption of miRNA mediated gene regulation plays a role in cancer development [33]. It is thus clear that not only the absence of, but also downregulation of *Dicer *results in disruption of cellular activity.

The immune response to RSV in infants is primarily driven by the innate immune system until the point of maximal symptoms, at which time recruitment of the cellular immune response hastens viral clearance [17,41] and clinical improvement. In leukocytes, *Dicer *downregulation in an anti-RSV setting would likely be most significant in the myeloid and natural killer cells, as they survey the lung environment, identify antigen and regulate the innate immune response to RSV. On recognition of viral antigen, myeloid dendritic cells recruit neutrophils and macrophages, and migrate to lymphoid tissue where they activate a cellular immune response [17,42]. Impairment of innate immune cellular function in association with *Dicer *downregulation in our patients may thus disturb the innate response to RSV and likely delay the recruitment of TH1 helper cells, which may also be dysfunctional [37].

The Dicer-mediated production of endogenous anti-viral miRNA may also be reduced in our patients. In an investigation of the role of Dicer in influenza, in-vitro knockdown of *Dicer *to a functional level of 30% in human alveolar cells resulted in increased influenza virus replication and greater apoptosis rates [21]. These were interesting results, and it is tempting to hypothesize that *Dicer *downregulation would similarly result in greater RSV viral load in our patients. This could result in increased apoptosis, greater activation of the immune response, more airways inflammation and therefore more severe disease. However, blood leukocytes and human alveolar cells are quite different cells types, and we therefore cannot assume that our results reflect *Dicer *expression levels in lung tissue. Our experiment was not designed to investigate the state of pulmonary epithelial cells in infants prior to RSV exposure, and such an experiment is currently not feasible due to ethical and practical issues.

RNAi as an anti-viral therapy currently receives much interest, and synthetic siRNAs with tailored activity against HIV, hepatitis B virus, human metapneumovirus and RSV are under development [24,43-45]. ALN-RSV01 (Alnylam Pharmaceuticals) is a synthesized siRNA with activity against the RSV N-protein [24]. In a recent trial, ALN-RSV01 was administered intra-nasally to adults before and after nasal inoculation with RSV. There was a significant anti-viral effect, with fewer patients RSV-culture positive in the ALN-RSV01 group compared to placebo [23]. In another trial, adult lung transplant recipients with proven RSV infection were randomized to nebulized ALN-RSV01 or placebo. The ALN-RSV01 group had a lower nasal RSV load, an improved symptom score, and a lower incidence of bronchiolitis obliterans syndrome, a known complication of RSV infection in this patient group [46]. Our findings suggest that infants susceptible to severe RSV infection may have a reduced capacity to produce anti-viral miRNA, strengthening the hypothesis that synthesized anti-RSV siRNA will have clinically relevant effects in lower respiratory RSV disease in infants. This is significant, given that after 5 decades there are still no safe and effective vaccines or treatments for RSV other than prophylaxis [16].

Our experiment was not designed to discover the cause of *Dicer *downregulation, and future experiments will be aimed at this. Genetic or epigenetic factors involving the *Dicer *gene or promoter may be at fault. In addition, dysregulation of other molecular systems can affect *Dicer *expression. A number of single nucleotide polymorphisms in genes of the immune system are associated with RSV [11], and we have previously shown downregulation of TNF receptor 25 in infants with RSV disease [25]. It is not clear how these factors would affect *Dicer *expression. However, one in vitro study showed that interferon-α downregulates and interferon-γ upregulates *Dicer *[47], suggesting that variations in response to stress (e.g.: birth) may explain our findings. Such differing stress responses could in themselves explain the predisposition for RSV disease.

## Conclusion

In summary, we demonstrate reduced *Dicer *expression at birth in the cord blood of infants with severe RSV disease, prior to RSV exposure. We theorize that this may predispose to RSV disease by disruption of both miRNA-associated gene regulation in leukocytes and direct anti-viral RNA interference mechanisms. We can thus add to the understanding of the pathophysiological processes in severe RSV disease.

## Abbreviations

cDNA: Complementary DNA; CI: Confidence interval; CPAP: Continuous positive airways pressure; Ct: Cycle threshold; ΔΔCt: Normalized and calibrated Cycle threshold; DNA: Deoxyribonucleic acid; dsRNA: Double-stranded RNA; GP: General practitioner (primary care physician); IQR: Inter-quartile range; M-PER: Mammalian Protein Extraction Reagent; miRNA: Micro RNA; mRNA: Messenger RNA; No-RT: Without reverse transcriptase; NPA: Nasopharyngeal aspirate; pCO2: Capillary partial pressure of carbon dioxide; PCR: Polymerase chain reaction; qPCR: Quantitative PCR; RIN: RNA integrity number; RISC: RNA-induced silencing complex; RNA: Ribonucleic acid; RNAi: RNA interference; RSV: Respiratory syncytial virus; RT-PCR: Reverse transcriptase PCR; S.D.: Standard deviation; siRNA: Small interfering RNA; TLDA: TaqMan low density array; TNF: Tumor necrosis factor; URTI: Upper respiratory tract infection

## Competing interests

The authors declare that they have no competing interests.

## Authors' contributions

HOF was responsible for collection of umbilical cord samples for analysis and identification of patients. TN and BN were responsible for laboratory work. CI was responsible for data analysis and statistical calculations. All authors were involved in conceptual design and planning of the qPCR analysis, manuscript preparation, interpretation of results, and have approved the final manuscript.

## Pre-publication history

The pre-publication history for this paper can be accessed here:

http://www.biomedcentral.com/1471-2334/11/59/prepub
